# Potential of plant growth promoting bacterial consortium for improving the growth and yield of wheat under saline conditions

**DOI:** 10.3389/fmicb.2022.958522

**Published:** 2022-09-29

**Authors:** Muhammad Yahya Khan, Sajid Mahmood Nadeem, Muhammad Sohaib, Muhammad Rashid Waqas, Fahad Alotaibi, Liaqat Ali, Zahir Ahmad Zahir, Fahad N. I. Al-Barakah

**Affiliations:** ^1^Sub-Campus Burewala-Vehari, University of Agriculture, Faisalabad, Pakistan; ^2^Department of Soil Sciences, College of Food and Agricultural Sciences, King Saud University, Riyadh, Saudi Arabia; ^3^Institute of Soil and Environmental Sciences, University of Agriculture, Faisalabad, Pakistan

**Keywords:** salinity, rhizobacteria, multi-strain, wheat, tolerance

## Abstract

Owing to inconsistent results of a single bacterial strain, co-inoculation of more than one strain under salinity stress could be a more effective strategy to induce salt tolerance. Co-inoculation of more than one bacterial strain could be more effective due to the presence of several growths promoting traits. This study was conducted to evaluate the effectiveness of multi-strains bacterial consortium to promote wheat growth under salinity stress. Several plant growth promoting rhizobacteria (PGPR) had been isolated and tested for their ability to grow in increasing concentrations of sodium chloride (NaCl). Those rhizobacterial strains having tolerance against salinity were screened to evaluate their ability to promote wheat growth in the presence of salinity by conducting jar trials under axenic conditions. The rhizobacteria with promising results were tested for their compatibility with each other before developing multi-strain inoculum of PGPR. The compatible PGPR strains were characterized, and multi-strain inoculum was then evaluated for promoting wheat growth under axenic conditions at different salinity levels, i.e., 2.1 (normal soil), 6, 12, and 18 dS m^–1^. The most promising combination was further evaluated by conducting a pot trial in the greenhouse. The results showed that compared to a single rhizobacterial strain, better growth-promoting effect was observed when rhizobacterial strains were co-inoculated. The multi-strain consortium of PGPR caused a significant positive impact on shoot length, root length, shoot fresh weight, and root fresh weight of wheat at the highest salinity level in the jar as well as in the pot trial. Results showed that the multi-strain consortium of PGPR caused significant positive effects on the biochemical traits of wheat by decreasing electrolyte leakage and increasing chlorophyll contents, relative water contents (RWC), and K/Na ratio. It can be concluded that a multi-strain consortium of PGPR (*Ensifer adhaerens* strain BK-30, *Pseudomonas fluorescens* strain SN5, and *Bacillus megaterium* strain SN15) could be more effective to combat the salinity stress owing to the presence of a variety of growth-promoting traits. However, further work is going on to evaluate the efficacy of multi-strain inoculum of PGPR under salt-affected field conditions.

## Introduction

In the soil environment, a plant faces both biotic and abiotic stresses, which cause a harmful impact on plant growth and development. In many areas of the world, including Pakistan, salinity is one of the most serious problems hampering agricultural production. About 40,000 ha of land is lost annually to cultivation due to salinity, and therefore causes a significant impact on the country’s economy (Aslam and Prathapar, 2006; Qadir et al., 2014). Salinity is considered a major cause of desertification and effect almost one-fourth of the world’s cultivated land (Machado and Serralheiro, 2017).

Under salinity stress, the plant faces a nutritional and hormonal imbalance as well as ion toxicity, and a huge amount of energy is required to modify the environment to accommodate the plant. Increased concentration of Na^+^ caused an increased uptake of Na^+^ and a decrease in K^+^ and Ca^2+^ contents of the plant (Abrar et al., 2020; Ndiate et al., 2021, 2022). Similarly, a significant quantity of ethylene produced under stress can damage the plants due to its negative impact on roots growth, and it can also cause epinasty and premature senescence (Nadeem et al., 2010). Soil microbial functioning has also been significantly affected by the influence of soil salinity stress (Liu et al., 2019).

Many efforts have been channelized to understand the mechanisms of stress tolerance and to enhance plant resistance against salinity stress. Among these, an environmentally sound and cost-effective option is the use of beneficial microbes for providing assistance to plants (Nazli et al., 2020). These beneficial bacteria including plant growth promoting rhizobacteria (PGPR) have been widely reported to help crop plants in various kinds of stresses under varying environmental conditions (Khan et al., 2013; Raza et al., 2021; Nadeem et al., 2022; Rafique et al., 2022). These PGPR protect the plant from the negative impact of salinity through a number of their direct and indirect mechanisms and therefore enable it to withstand stress environment (Zahir et al., 2009, 2019; Fu et al., 2010; Glick, 2013; Nadeem et al., 2016; Ullah et al., 2017; Al-Barakah and Sohaib, 2019; Rafique et al., 2021; Saeed et al., 2021; Singh P. et al., 2022; Upadhyay and Chauhan, 2022; Upadhyay et al., 2022). However, under certain stresses, the results obtained in axenic conditions could not be reproduced in the field (Smyth et al., 2011). This might be occurred due to the low quality of the inoculums and/or the inability of the bacteria to compete with the indigenous population (Catroux et al., 2001). The reasons for the poor performance of agricultural bio-inocula in natural environments and in the rhizosphere of host plants could be the use of a single bacterial strain (Van Veen et al., 1997). Therefore, instead of using a single bacterial strain with single trait/multiple traits for inoculation of seeds/seedlings, multiple microbial consortia could be used for multiple benefits. The preparation of a multi-strain bacterial consortium by keeping in view their compatibility enables the bacterial strain to communicate synergistically with each other by decreasing inhibitory products and providing more balanced plant nutrition improve plant growth and development in variable environments (Wani et al., 2007). In certain cases, single strain inoculation fails to compete with indigenous soil microflora due to its low root colonization percentage and poor survival efficiency (Bashan, 1998; Elkoca et al., 2010) significant positive results are not obtained (Smyth et al., 2011). Compared to a single strain, a multi-strain consortium improves plant growth through the cumulative effect of various mechanisms adopted by different microbial strains (Khan and Zaidi, 2007). Jha and Saraf (2012) observed that the growth of the Jatropha plant (*Jatropha curca*) improved maximally in greenhouse and field experiments when three strains were applied together. Similarly, the consortia of three strains gave the best performance in terms of growth parameters of *Lycopersicum esculentus* (Nicolaus et al., 1999). They demonstrated that the use of combined biofertilizers containing consortia of bacteria was an excellent inoculant for the growth performance of the plants.

As far as growth under stress environment is concerned, Annapurna et al. (2011) studied the effectiveness of PGPR separately and in combination for reducing the impact of salinity on wheat growth. They found that single and dual inoculations of PGPR strains showed variations in their effect to enhance the crops’ tolerance to salt. The bacterial consortium was more effective in inducing salinity tolerance in wheat plants. They considered it as an acceptable and environment-friendly technology to improve plant performance and development under stress environments. The results of their study indicated that co-inoculation with *Bacillus subtilis* and *Arthrobacter* sp. could alleviate the adverse effects of soil salinity on wheat growth. The use of multi-strain bacterial consortia in agriculture has become an area of interest because of its positive impact on plant growth compared to single inoculation. The positive interactions between rhizobacteria could be helpful in better colonization and combating stress-induced negative impact.

Some recent studies show that the effectiveness of bacterial consortia for enhancing plant growth over a single bacterial strain (Moronta-Barrios et al., 2018; Chandra et al., 2019; Olanrewaju and Babalola, 2019). However, most of this work has been conducted under normal conditions or plant protection against biotic stress. Furthermore, some preliminary studies also indicate that multi-strain bacterial consortium offers more sustainable plant growth improvement (Khan et al., 2017; Irfan et al., 2019; Sohaib et al., 2020). Limited work has been conducted under salinity stress therefore the application of multi-strains bacterial consortium over single inoculation could be an effective approach for reducing the harmful impact of salinity stress on plant growth, which is one of the major growth limiting factors in arid and semi-arid regions. Therefore, this study was undertaken with the objective to evaluate the effectiveness of multi-strain bacterial consortium for improving the growth and development of wheat under salt stress condition.

## Materials and methods

### Isolation of plant growth promoting rhizobacteria and salinity tolerance assay

A total of 55 samples of rhizosphere soil samples were collected from the salt-affected fields of wheat (30°14′35.9”N 72°43′19.7” E). The rhizosphere sample was collected by following the procedure adopted by Khan et al. (2017). The rhizosphere soil adhering to the roots was collected and soil suspension obtained was used to isolate rhizobacteria by dilution plate method by using DF minimal salt medium (Dworkin and Foster, 1958) containing ACC as a sole source of nitrogen (Jacobson et al., 1994). The isolated rhizobacterial strains were purified by further 2–3 times streaking on freshly prepared DF minimal salt medium and pure colonies were used for further screening.

A total of 50 isolated bacterial strains including two previously characterized PGPR strains, i.e., *Pseudomonas fluorescens* strain SN5 (Accession Number; JN858098) and *Bacillus megaterium* strain SN15 (Accession Number; JN858088) were examined for their ability to tolerate salinity. For this purpose, tryptic soya broth (TSB) with 2.5, 5, and 7.5% sodium chloride (NaCl) were prepared and inoculated with respective strains. For each isolate, triplet test tubes at each salinity level were inoculated with 0.5 mL inoculum of OD 0.5 (>10^8^ cell mL^–1^) and incubated for 72 h at 28 ± 1°C, shaking at 100 rpm. PGPR isolates showing high optical density (OD) at 600 nm under salinity were considered salinity tolerant.

### Screening of plant growth promoting rhizobacteria for promoting wheat growth under salinity stress

The 20 bacterial isolates selected on the basis of salinity tolerance assay were evaluated for their growth promoting potential For this purpose, a jar experiment was conducted under axenic conditions. Four salinity levels, i.e., original, 6, 12, and 18 dS m^–1^ were used. Salinity levels were developed by NaCl salt in sterilized half-strength Hoagland solution (Machado and Serralheiro, 2017). Two sterilized filter paper sheets were soaked and saturated with respective inoculum. Four surface-sterilized wheat seeds were sandwiched between these two sterilized filter papers, which were rolled and placed in the glass jars (Asghar et al., 2002). Seeds were surface sterilized by dipping in 70% ethanol for 1 min and 3.5% sodium hypochlorite for 3–5 min and followed by 3–4 washings with autoclaved distilled water (Long et al., 2008). Jars were arranged using a completely randomized design with four replications for each treatment. Jars were placed in a growth chamber at 25 ± 1°C, adjusted to 16 h of light (supplied by a mixture of incandescent and fluorescent lamps at an intensity range of 200–225 μE m^–2^s^–1^), and an 8 h dark period. After 3 weeks, four plants were harvested and data regarding shoot/root length, shoot\root fresh weight, and shoot/root dry weight were recorded.

### Compatibility test

On the basis of the results of the axenic trial, 10 most promising strains (DG-18, DG-34, BK-6, BK-30, BK-46, BK-50, UA-3, UA-46, SN5, and SN15) were tested for their compatibility with each other in all possible combination by cross streak assay on nutrient agar medium (Raja et al., 2006). One bacterial strain was streaked on the solidified nutrient agar plate and incubated at 28 ± 1°C for 24 h for growth. The counter bacterial strain was streaked vertically to the growth of the already streaked bacterial strain. The plates were incubated at 28 ± 1°C for 48 h and bacterial growth was observed. The growth suppression of counter bacterial strain by already streaked bacterial strain was considered as un-compatible and mixing of bacterial growth of both strains was considered as compatible. Among these strains, six strains (DG-34, BK-30, UA-3, UA-46, SN5, and SN15) showed compatibility when used in various combinations.

### Characterization of compatible strains

The six strains showed compatibility in various combinations and were characterized by their plant growth promoting traits. The ACC-deaminase activities of the strains were measured quantitively by determining the amount of α-ketobutyrate produced when the enzyme cleaved ACC into ammonia and α-ketobutyrate according to the method of Penrose and Glick (2003). All measurements were carried out in triplicate and ACC-deaminase activity was calculated as μmol α-ketobutyrate mg^–1^ protein h^–1^. Exopolysaccharides production was measured qualitatively according to the method of Nicolaus et al. (1999). For this purpose, strains were grown in a culture medium, and supernatants were collected by centrifuging at 1,000 rpm. The formation of the precipitate by the addition of cold absolute ethanol dropwise was an indication of the production of exopolysaccharides.

Phosphate solubilization was determined qualitatively by using the National Botanical Research Institute (NBRI) growth medium according to Mehta and Nautiyal (2001). Individual bacterial isolates were cultured, and spot inoculated in the center of the agar plates. Colonies showing the formation of clear zones after incubating at 28°C for 5 days were considered positive for phosphate solubilization, and the diameter of the clear halo zone was measured. Siderophore production of the strains was assayed qualitatively according to the universal method of Schwyn and Neilands (1987). Fresh cultures of each strain were inoculated into a modified MM9 medium (low iron medium). The cultures were centrifuged after incubation at 28°C for 48 h. The supernatant was then mixed with the chrome azurol S (CAS) solution. The change of color from blue to orange indicated the presence of siderophores in the solution.

Indole acetic acid production of the selected six isolates was determined colorimetrically as used by Sarwar et al. (1992). For this purpose, 5 mL of filter sterilized 0.5% l-tryptophan solution was added to the flask and inoculated with a particular strain, and then incubated at 28°C for 48 h on a horizontal shaker at 100 rev min^–1^. Un-inoculated control was also kept for comparison. After incubation, the contents were filtered, and 3 mL of the filtrate was mixed with 2.0 mL of Salkowski reagent and allowed to stand for 30 min for color development. Auxin compounds expressed as IAA equivalents were determined by spectrophotometer (Sarwar et al., 1992).

### Screening of multi-strain consortium for promoting wheat growth under salinity stress

Six rhizobacterial strains showed compatibility with each other and were used in various combinations as co-inoculation and multi-strain inoculum. These combinations were DG-34 × BK30, UA-3 × UA-46, UA-3 × SN5, UA-46 × SN15, BK-30 × SN5, UA-3 × SN15, BK-30 × SN15, UA-3 × SN15 × UA-46, and BK-30 × SN5 × SN15. These combinations were tested for evaluating their ability to promote wheat growth under salinity stress. The inoculum of each strain was prepared as described above. For inoculating the sterilized wheat seed with multi-strain inoculum, broth cultures of desired bacterial strains were mixed in equal proportion and vortexed for 5 min to ensure homogenized cell density of different bacterial strains before seed dipping. Surface sterilized seeds of wheat were placed in sterilized Petri plates enfolded in two sheets of filter paper moistened with sterilized distilled water. These Petri plates were incubated at 25 ± 1°C for germination in an incubator under darkness for 2 days. Fully sprouted seeds of wheat and maize were dipped for 10 min in broth culture of the required bacterial population (10^7^–10^8^ cfu mL^–1^). Four salinity levels, i.e., 0, 6, 12, and 18, as described above were prepared.

Salinity levels were developed by NaCl salt in the sterilized Hoagland solution (Machado and Serralheiro, 2017) of half-strength. The actual salinity levels obtained after developing salinity were 0.8, 6.2, 12.5, and 17.4 dS m^–1^. Jars were arranged using completely randomized design with four replications for each treatment. Jars were placed in a growth chamber at 25 ± 1°C, adjusted to 16 h light (supplied by a mixture of incandescent and fluorescent lamps at an intensity range of 200–225 μE m^–2^s^–1^) and an 8 h dark period. After 3 weeks, plants were harvested and data regarding shoot/root length, shoot\root fresh weight, and shoot/root dry weight were recorded and analyzed statistically.

### Root colonization assay

The root colonization ability of bacterial isolates was determined by the modified method of Simons et al. (1996). After 1 week of starting the jar experiment, the plant from one replication was harvested and rinsed with distilled water, and blotted to dryness. The root tips (0.2 g) were removed and put into a flask containing 5 mL of sterilized distilled water. The root samples were shaken vigorously for 30 min using an orbital shaker (Memmert, Schwabach, Germany). Serial dilutions of bacterial suspensions were prepared and 1 mL of each dilution was spread on already prepared tryptic soya agar plates (TSA). All treatments in the colonization experiment were replicated three times. The average number of colony-forming units (cfu) per gram root was determined after 24 h of incubation at 28 ± 2°C using a digital colony meter (J.P Selecta, Barcelona, Spain).

### Pot trial

From the results of the axenic trial, the two combinations (UA-3 + SN15 + UA-46 and BK-30 + SN5 + SN15) were selected on the basis of their better performance by promoting growth at the highest salinity level. A third combination having a consortium of two strains, i.e., UA-46 + SN15 was also selected for comparison. There were four treatments, i.e., T_1_ (Control), T_2_ (UA-46 + SN15), T_3_ (UA-3 + SN15 + UA-46), and T_4_ (BK-30 + SN5 + SN15). For developing a multi-strain consortium, a fresh inoculum of each strain was prepared in sterilized broth separately as described earlier. Before inoculating the wheat seeds, an optical density (OD) of 0.5 was developed to maintain a population density of 10^7^–10^8^ cfu mL^–1^.

For conducting a pot trial, the soil was ground and passed through a 2 mm sieve. The electrical conductivity (EC) of soil was 2.1 dS m^–1^. The required salinity levels, i.e., 6, 12, and 18 dS m^–1^ were developed by thoroughly mixing the calculated amount of NaCl in the sieved soil.

After developing salinity, each pot was filled with 12 kg of soil and was arranged according to a complete randomized design with three replications. For inoculating wheat seed, a consortium of three strains were prepared by mixing equal proportion (at a ratio of 1:1:1 having 4 mL inoculum of each strain) and a consortium of two strains was prepared by mixing equal proportion (at a ratio of 1:1 having 6 mL inoculum of each strain). Surface-disinfected wheat seeds were inoculated with sterilized (autoclaved) peat mixed with 10% sterilized sugar solution (1:1 w/w inoculum to peat ratio).

Five wheat seeds were sown in each pot at an equal distance. The pots of each treatment were arranged randomly and NPK at the rate of 120:90:60 kg ha^–1^ were used as urea, diammonium phosphate, and muriate of potash fertilizers. Good quality canal water (EC = 0.7 dS m^–1^, SAR = 0.5 (mmol L^–1^)^1/2^ and RSC = 0.05 mmolc L^–1^) meeting the irrigation quality criteria of the crops (Ayers and Westcot, 1985) was used for irrigation. Twenty days after germination, a uniform plant population of two seedlings per pot was maintained by thinning. In all these experiments, wheat crop variety Faisalabad-2008 (Approved by Punjab Seed Corporation (PSC), Pakistan in September, 2008) was used as a test wheat variety.

### Data collection and physiological analysis

Seventy days after sowing, plant leaf samples were collected for analyzing some chemical and biochemical parameters like sodium, potassium, chlorophylls (a and b), relative water content (RWC), membrane stability, and proline contents. At maturity, data regarding plant height, number of tillers per pot, number of spikelets per spike, grain yield per pot, root length, and 1,000-grain weight were collected. Wheat shoot and grain samples were analyzed for nitrogen and phosphorus content.

For determining chlorophyll contents, 0.5 g of leaf sample was collected from each treatment and homogenized with 80% acetone (v/v). The homogenate was filtered through filter paper. The absorbance of the resulting solution was read by spectrophotometer at 663 and 645 nm for chlorophylls a and b, respectively (Arnon, 1949). Chlorophylls a and b were calculated using the following formula and results were mentioned as total chlorophyll content, i.e., Chlorophyll a + b:


Chl`a(mg/gfreshweight)′

$$=\left[\left\{\left(12.7\times \text{OD}\,663\right)-\left(2.69\times \text{OD}\,645\right)\right\}\times \frac{\text{V}}{1000}\times \text{W}\right]$$

Chl`b(mg/gfreshweight)′

$$=\left[\left\{\left(22.9\times \text{OD}\,645\right)-\left(4.68\times \text{OD}\,663\right)\right\}\times \frac{\text{V}}{1000}\times \text{W}\right]$$

where,


OD663=OpticalDensityatawavelengthof663⁢nm

OD645=OpticalDensityatawavelengthof645⁢nm

V=Volumeofthesample,

W=Weightoffreshtissue

RWC was determined by using the following formula as described by Mayak et al. (2004):


(1) $$\text{RWC}\left(\%\right)=\frac{\left(\text{FW}-\text{DW}\right)}{\left(\text{FTW}-\text{DW}\right)}\times 100$$

where,


RWC=RelativeWaterContents

FW=Freshweight

DW=Dryweight

FTW=Fullyturgidweight

The fully turgid weight (FTW) is defined as the weight of the leaf after it was held in 100% humidity conditions in the dark at 4°C for 48 h.

Membrane stability was determined by calculating electrolyte leakage according to the method described by Lutts et al. (1996). One young leaf was collected from two plants from each treatment and washed thoroughly with deionized water to remove surface-adhered electrolytes.

The samples were placed in closed vials with deionized water and incubated on a rotary shaker for 24 h at 25°C. The EC of the solution (Lt) was determined by a conductivity meter. Samples were then autoclaved at 120°C for 20 min and the final EC of the solution (L0) was measured after cooling. The electrolyte leakage was determined by the formula:


$$\text{EL}\left(\%\right)=\left(\frac{\text{Lt}}{\text{L}\,0}\right)\times 100$$

Free proline content was determined according to the method described by Bates et al. (1973). One gram leaf sample was homogenized in 3% sulphosalyclic acid and then filtered through Whatman filter paper No. 2. After the addition of acid ninhydrin and glacial acetic acid, the mixture was heated at 100°C for 1 h in a water bath and the reaction was stopped using an ice bath. The mixture was extracted with toluene and the absorbance was read at 520 nm. Proline concentration was determined using a standard curve and was expressed as μmol g^–1^.

### Plant analyses

The grain samples of wheat were first digested for determining nitrogen and phosphorus according to the method of Wolf (1982). The sample was digested with conc. H_2_SO_4_ and H_2_O_2_ (35% A. R. grade extra pure) at 350°C. After digestion, nitrogen was determined by the Kjeldahl method. The phosphorus-digested sample was mixed with Barton reagents and phosphorus was determined by spectrophotometer using a standard curve.

### Identification of rhizobacterial strains

Rhizobacterial isolates were identified based on the *16S ribosomal RNA* (*16S rRNA*) gene sequencing. Pure colonies of efficient bacterial isolates (DG-34, BK-30, UA-3, and UA-46) were sent to Macrogen Inc., Korea for getting sequences of the target gene. The BlastN tool of the NCBI database^1^ was used to compare with the known nucleotide sequences of *16S rRNA* genes. Bacterial isolates were given names as *Atlantibacter hermannii* strain DG-34 (Accession Number; OP204096), *Ensifer adhaerens* strain BK-30 (Accession Number; OP204097), *Stenotrophomonas maltophilia* strain UA-3 (Accession Number; OP295490), and *Atlantibacter hermannii* strain UA-46 (Accession Number; OP295491) based on the maximum similarity with known bacteria.

### Statistical analyses

Standard errors (SE) were estimated using Microsoft Excel 2016^®^ (Microsoft Cooperation, USA), and the Student’s *t*-test was applied for the level of significance (*P* < 0.05). Data were statistically analyzed using the statistical software (Statistix-8.1^®^; Analytical Software, Tallahassee, USA).

## Results

### Salinity tolerance of isolated strains

**FIGURE 1:**
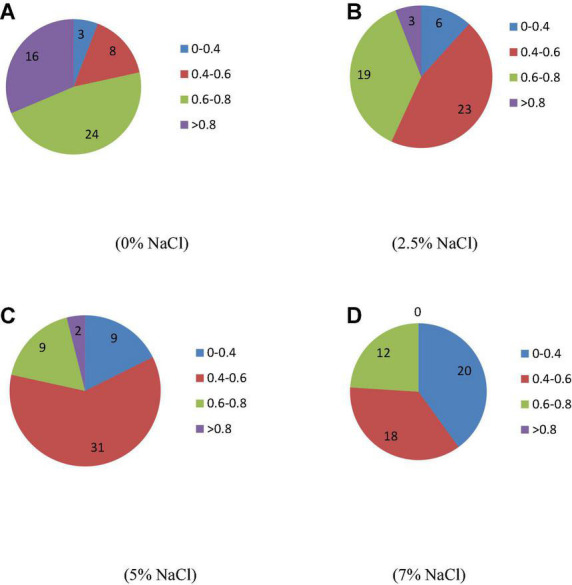
Impact of different salinity levels on optical density (600 nm) of isolates. **(A–D)** Different levels of salinity.

A total number of 50 isolated strains were evaluated for their ability to tolerate saline conditions. The results show that strains had great variability regarding their growth under saline conditions. The optical density values of certain strains remained very low with increasing levels of salinity that show their limited growth in the saline environment (Figure 1). At normal condition (0% NaCl), 26 strains (52%) showed 6–8 optical density and 16 strains (32%) showed optical density value of more than 8. With increasing salinity, the optical density decreased. At 2.5% NaCl, 9 (18%) and 31 (62%) strains showed optical density values in the range of 6–8 and > 8, respectively. At 5% NaCl, only three strains (6%) showed optical density value > 8. However, no strain showed this optical density value (> 8) at saline media containing 7.5% NaCl. Twenty strains, i.e., 40% showed optical density values in the range of 6–8. This indicates the ability of these strains to tolerate high salinity.

### Screening of plant growth promoting rhizobacteria for promoting wheat growth under salinity stress

Out of these 50 strains used to evaluate their growth in a saline environment, 20 strains were selected based on their ability to tolerate salinity. These 20 strains were further evaluated for their efficacy to promote wheat growth by conducting jar trials under salinity stress conditions. The data regarding shoot and root parameters were recorded.

Our results indicated that bacterial inoculation enhanced the shoot length at all salinity levels (Supplementary Table 1). This impact was more pronounced at high salinity levels 12 and 18 dSm^–1^. At 6 dSm^–1^ strain, DG-48 showed a maximum increase in shoot length by 55%, and at this level, strains UA-46, UA-1, and BK-50 increased shoot length up to 47, 48, and 42%. Similarly, at salinity level 12 dSm^–1^ strains DG-18 and UA-3 gave maximum shoot length and increased the shoot length up to 69 and 61%. At higher salinity (18 dSm^–1^) maximum shoot length was showed by strain DG-18 followed by strain UA-3, which increased shoot length up to 140% as compared to the un-inoculated control. Data predicted in Supplementary Table 2 showed that strains DG-52 and BK-50 increased shoot fresh weight up to 13% at 6 dSm^–1^, followed by the strains UA-1, BK-23, DG-18, and DG-48. At 12 dSm^–1^, UA-1 gave the best results and caused an increase in shoot fresh weight of wheat seedling up to 18%, and strains DG-12, BK-50, BK-28, and UA-44 were the next most effective strains. Strains BK-6 and BK-30 showed good results at EC 18 dSm^–1^ and increased shoot fresh weight up to 60% compared to the un-inoculated control. The data regarding shoot dry weight showed that at 6 dSm^–1^, DG-48 performed better and increased shoot dry weight up to 96%. At 12 and 18 dSm^–1^, strains SN5 and UC-14 increased shoot dry weight by 100 and 200% over un-inoculated control followed by UA-44 and UA-46 (Supplementary Table 3).

Supplementary Table 4 indicated that strains had variable responses regarding root length. All strains increased the root length of wheat in the case where no salt was added (normal). At 6 dSm^–1^, the highest root length was observed by BK-46, while strain DG-18 exhibited a maximum increase in root length at EC level 12 dS m^–1^. At higher salinity levels, i.e., 18 dS m^–1^ strain BK-33 was found to increase root length up to 177%. Inoculation of wheat seeds caused a significant increase in root fresh weight at different salinity levels (Supplementary Table 5). At 6 dS m^–1^, strains UA-1 and SN5 resulted in a maximum increase in fresh weight that was 29 and 28% higher than the uninoculated control. BK-46 caused a 20% increase in root fresh weight at salinity level 12 dS m^–1^. At high salinity level (18 dS m^–1)^, strains UA-44 and SN15 performed better and increase root fresh weight by 45 and 42%. The effect of bacterial inoculation on root dry weight is presented in Supplementary Table 6. At 6 dS m^–1^, the strain SN15 caused an increase up to 46%, while an increase of 146% was observed due to strain DG-8 at EC level 12 dS m^–1^. At higher salinity level, i.e., 18 dS m^–1^, maximum root dry weight was shown by DG-8, DG-34, BK-30, UA-44, and SN15, which cause an increase up to 166% as compared to their respective controls.

### Multi-strain grouping

Out of 20 strains tested in the jar experiment, the strains that gave promising results were examined for their compatibility with each other. On the basis of the compatibility test, six strains were selected and then used in different combinations to prepare a multi-strain bacterial consortium for enhancing salinity tolerance in wheat.

### Screening of multi-strain consortium for promoting wheat growth under salinity stress

A further jar experiment was conducted to evaluate the efficacy of bacterial consortium for promoting the growth of wheat grown under salinity stress conditions. Obtained results showed that multi-strain inoculum promoted wheat growth significantly more than dual inoculation.

**FIGURE 2:**
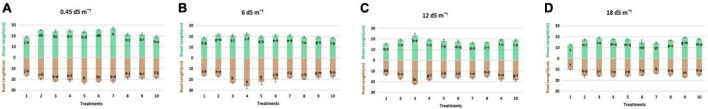
Effect of multi-strains inoculum on shoot length (cm plant^–1^) and root length (cm plant^–1^) of wheat in the presence of salinity under the axenic condition. **(A–D)** Different levels of salinity.

**FIGURE 3:**
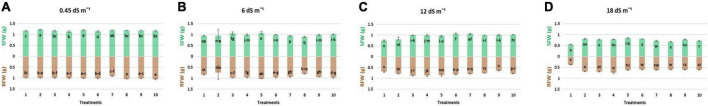
Effect of multi-strains inoculum on shoot fresh weight: SFW (g plant^–1^) and shoot fresh weight: RWF (g plant^–1^) of Wheat in the presence of salinity under the axenic condition. **(A–D)** Different levels of salinity.

**FIGURE 4:**
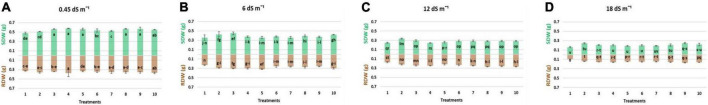
Effect of multi-strains inoculum on shoot dry weight: SDW (g plant^–1^) and root dry weight: RDW (g plant^–1^) of wheat in the presence of salinity under the axenic condition. **(A–D)** Different levels of salinity.

Results regarding the shoot length of wheat seedlings presented in Figure 2 demonstrated that salinity decreased the shoot length compared to normal (non-saline). The reduction in shoot length was more at high salinity levels (12 and 18 dS m^–1^) as compared to low-salinity level. However, inoculation with different strains enhanced the shoot length at all salinity levels, i.e., normal, 6, 12, and 18 dS m^–1^. Under axenic conditions, shoot length was significantly improved when the strains were used in different combinations. At 6 dS m^–1^, the dual combinations of UA-3 × SN5 and DG-34 × BK-30 enhanced shoot length up to 19 and 17%. At high salinity levels, i.e., 12 dS m^–1^ the combination UA-3 × UA-46 increased the shoot length by 49%. However, at 18 dS m^–1^, multi-strain inoculation was used, and the combination UA-3 × SN15 × UA-46 increased the shoot length up to 51%. Inoculation with strains improved the shoot fresh weight at low (normal and 6 dS m^–1^) and high (12 and 18 dS m^–1^) salinity levels as compared to respective uninoculated control (Figure 3). Shoot fresh weight significantly increased when combinations of UA-46 × SN15 were used, which increased fresh weight up to 12% at 6 dS m^–1^. At 12 dS m^–1^, the combinations of BK-30 × SN5 and at 18 dS m^–1^, BK-30 × SN5 × SN15 caused a 41 and 47% increase in shoot fresh weight, respectively. The effect of various salinity levels on shoot dry weight of wheat seedlings and comparative efficacy of co-inoculation and multi-strain inoculation for improving shoot weight is presented in Figure 4. Results showed that the response of co-inoculation and multi-strain inoculation was significantly better than the un-inoculated control. An increase of 25% was observed in the combination of UA-3 × UA-46 when EC was 6 dS m^–1^. At higher salinity, the combination of UA-3 × SN15 × UA-46 increased shoot dry weight up to 56% as compared to the un-inoculated control.

Root length was increased due to inoculation with different strains at low (normal) and high (12 and 18 dS m^–1^) salinity levels (Figure 2). At normal, the combinations of UA-46 × SN15 and BK-30 × SN5 enhanced root length up to 39 and 34%, respectively. The combination of UA-3 × SN5 caused an increase up to 57% at 6 dS m^–1^. At higher salinity levels (i.e., 12 and 18 dS m^–1^), the co-inoculation of UA-3 × SN15 × UA-46 exhibited an increase up to 61 and 83%. Data given in Figure 3 indicate that different salinity levels (6, 12, and 18 dS m^–1^) had posed a significant reduction in root fresh weight of wheat seedlings as compared to normal. It is clearly evident from the results that inoculation reduced the salinity impact on wheat seedlings and improved the root fresh weight as compared to their respective controls. All co-inoculation and multi-strain inoculation caused an improvement in root fresh weight. At 6 dS m^–1^, the combination of UA-46xSN15 caused an increase of up to 22%. At 12 dS m^–1^, the combination of BK-30 × SN5 × SN15 posed an increase up to 100%, whereas at 18 dSm^–1^, UA-3 × UA-16 × UA-46 caused up to 72% increase in root fresh weight. Salinity stress significantly decreased the root dry weight at all salinity levels (6, 12, and 18 dS m^–1^) as compared to normal (Figure 4). However, various strains well supported the wheat seedlings to tolerate the salinity stress by improving root dry weight. An increase of 39% was observed when co-inoculation of UA-46 × SN15 was done at 6 dS m^–1^. At 12 dS m^–1^, the combination of BK-30 × SN5 × SN15 showed statistically similar results and caused up to a 58% increase in root dry weight as compared to their respective un-inoculated controls. The combination of BK-30 × SN5 × SN15 gave significant results and caused up to a 180% increase at 18 dSm^–1^.

### Plant growth promoting characteristics of strains

The selected strains were characterized by certain growth promoting traits (Table 1). The results of characterization assays showed that all six strains (DG-34, BK-30, UA-3, UA-46, SN5, and SN15) were positive for ACC-deaminase. UA-16 was highest in ACC-deaminase activity followed by UA-3 and UA-46. DG-34 showed the lowest ACC-deaminase activity compared to other strains. All strains for positive for phosphate solubilization and exopolysaccharides activity except DG-34, which was due to the lack of exopolysaccharides production. DG-34, UA-3, SN5, and SN15 were found positive for siderophores production.

**TABLE 1:** Characterization and identification of the rhizobacterial strains.

PGPR strain	Identification	Accession number (NCBI database)	Exopolysaccharides activity	ACC-deaminase activity (μ mol kg^–1^ h^–1^)	Phosphate solubilization	Siderophores	Indole acetic acid (μ g mL^–1^)
DG-34	*Atlantibacter hermannii*	OP204096	+	96	+	+	13.4
BK-30	*Ensifer adhaerens*	OP204097	+	102	+	–	15.2
UA-3	*Stenotrophomonas maltophilia*	OP295490	+	176	+	+	12.7
UA-46	*Atlantibacter hermannii*	OP295491	+	135	+	–	17.2
SN5	*Pseudomonas fluorescens*	JN858098	+	110	+	+	16.3
SN15	*Bacillus megaterium*	JN858088	+	221	+	+	15.5

### Root colonization assay

Data regarding root colonization showed that all selected strains efficiently colonized wheat roots (Table 2). Maximum root colonization was observed in the case of SN15, which was 5.21 × 10^5^ followed by UA-3 (3.45 × 10^5^) and SN5 (3.31 × 10^5^). UA-46 and BK-30 were the next effective strains regarding root colonization ability. Strain DG-34 showed less root colonization ability (3.46 × 10^4^) compared to other strains.

**TABLE 2:** Root colonization ability of bacterial strains.

Bacterial strains	DG-34	BK-30	UA-3	UA-46	SN5	SN15
Root colonization (cfu g^–1^)	3.46 × 10^4^	4.10 × 10^4^	3.45 × 10^5^	4.61 × 10^4^	3.31 × 10^5^	5.21 × 10^5^

### Pot trial

The multi-strain bacteria caused a significant impact on the growth and yield parameters of wheat. The results of the pot trail are described below.

Data in Table 3 show that the plant height of wheat seedlings increased significantly due to inoculation with the multi-strain bacterial consortium. Maximum increase (up to 33% over un-inoculated control) in plant height was recorded by inoculation with T_4_ at EC 17.5 dS m^–1^, which was statistically similar to T_2_ and T_3_. High salinity level (17.5 dS m^–1^) affected the number of tillers to a great extent; however, inoculation with multi-strain consortia mitigate this negative effect (Table 3). A maximum number of tillers at this EC (17.5 dS m^–1^) was obtained by T_4_, which was 128% more than the control. T_2_ and T_3_ were the next effective combinations. These two combinations were statistically similar to each other and caused up to an 86% increase in the number of tillers compared to the control.

**TABLE 3:** Effect of inoculation with multi-strain bacterial consortium on plant height (cm plant^–1^) and the number of tillers of wheat at different salinity levels in a pot trial.

Strain	Plant height				Number of tillers			
	Salinity (dS m^–1^)				Salinity (dS m^–1^)			
	2.1	6	12	18	2.1	6	12	18
T_1_	63.3 cd	60.3 de	49.7 g	42.0 h	4.67 bcd	4.67 bcd	3.33 de	2.33 e
T_2_	74.0 a	67.3 bc	60.7 de	53.7 fg	6.33 a	5.33 abc	4.33 cd	4.33 cd
T_3_	71.7 ab	68.0 bc	64.7 cd	55.0 f	6.33 a	5.33 abc	5.33 abc	4.33 cd
T_4_	70.7 ab	72.0 ab	62.0 d	56.0 ef	6.33 a	6.00 ab	5.33 abc	5.33 abc
LSD (5%)	5.164				0.656			

Means sharing the same letter(s) are statistically non-significant according to Duncan’s multiple range test (p < 0.05).

T_1_ (Control), T_2_ (UA-46 + SN15), T_3_ (UA-3 + SN15 + UA-46), T_4_ (BK-30 + SN5 + SN15).

Data regarding the number of spikelets per spike of wheat under salinity stress indicated that with an increase in salinity, the number of spikelets per spike of wheat decreased except for T_4_ at 12.1 dS m^–1^ (Table 4). At high salinity, i.e., 17.5 dSm^–1^ again T_4_ caused a significant increase in the number of spikelets over control, which was 35% more than the un-inoculated control. Data regarding root length revealed that in most of the cases, the statistically similar increase was observed by inoculation with selected PGPR strains that differed significantly from the un-inoculated control (Table 4). Consortium T_4_ performed better than other strains and caused 35, 44, and 66% increases over un-inoculated control at 5.8, 12.1, and 17.5 dS m^–1^, respectively. Inoculation with multi-strain bacterial consortium caused a significant increase in the grain yield of wheat compared to the un-inoculated control (Table 5). At higher salinity levels (12.1 and 17.5 dS m^–1^), T_4_ performed significantly better than other groups and increased grain yield up to 56 and 50%, respectively, over the un-inoculated control. Similarly, data regarding 1,000 grain weight at a high salinity level, i.e., 17.5 dSm^–1^ T_4_ again showed a maximum 1,000 grain weight that was 36% more than the un-inoculated control (Table 5).

**TABLE 4:** Effect of inoculation with multi-strain bacterial consortium on a number of spikelets per spike of wheat at different salinity levels in a pot trial.

Strain	Number of spikelets per spike				Root length			
	Salinity (dS m^–1^)				Salinity (dS m^–1^)			
	2.1	6	12	18	2.1	6	12	18
T_1_	17.3 abc	14.6 de	13.3 ef	11.3 f	18.7 bc	13.6 cdef	13.0 efg	8.00 g
T_2_	18.6 a	17.3 abc	15.3 cde	13.3 ef	21.0 a	17.3 bcde	14.6 cdef	12.0 fg
T_3_	18.3 ab	16.0 bcd	15.3 cde	14.3 de	20.3 ab	17.3 bcde	17.6 bcde	11.6 fg
T_4_	18.0 ab	15.0 cde	17.3 abc	15.3 cde	20.3 ab	18.3 bcd	18.7 bc	13.3 def
LSD (5%)	2.47				5.07			

Means sharing the same letter(s) are statistically non-significant according to Duncan’s multiple range test (p < 0.05).

T_1_ (Control), T_2_ (UA-46 + SN15), T_3_ (UA-3 + SN15 + UA-46), T_4_ (BK-30 + SN5 + SN15).

**TABLE 5:** Effect of inoculation with multi-strain bacterial consortium on grain yield (g pot^–1^) and 1,000-grain weight of wheat at different salinity levels in a pot trial.

Strain	Grain yield				1,000 grain weight			
	Salinity (dS m^–1^)				Salinity (dS m^–1^)			
	2.1	6	12	18	2.1	6	12	18
T_1_	11.87 b	9.67 c	6.00 ef	5.03 f	3.63 defg	3.40 g	2.91 h	2.69 h
T_2_	13.10 ab	12.27 b	9.07 cd	7.13 e	3.74 cde	3.82 bcde	3.66 def	3.42 fg
T_3_	14.60 a	12.93 ab	9.30 c	7.30 de	4.06 ab	4.11 a	3.68 de	3.57 efg
T_4_	14.70 a	12.73 b	9.37 c	7.56 d	3.96 abc	3.84 bcd	3.72 cde	3.66 def
LSD (5%)	1.827				0.253			

Means sharing the same letter(s) are statistically non-significant according to Duncan’s multiple range test (p < 0.05).

T_1_ (Control), T_2_ (UA-46 + SN15), T_3_ (UA-3 + SN15 + UA-46), T_4_ (BK-30 + SN5 + SN15).

### Physiological parameters

Data regarding chlorophyll contents in Table 6 revealed that inoculation effect was significant at different salinity levels. At low- and medium-salinity levels (5.8 and 12.1 dSm^–1^), T_3_ caused a maximum increase in chlorophyll content. Up to 127% increase in chlorophyll contents was observed with T4 at 17.5 dSm^–1^ followed by T_3_. As compared to higher salinity levels, no significant impact of inoculation was observed on electrolyte leakage at low salinity (Table 6). At 12.1 dS m^–1^, bacterial consortium T_4_ caused a maximum increase in membrane stability, i.e., 16% more than un-inoculated control. At 17.5 dS m^–1^, T_4_ performed better and increased the membrane stability of wheat leaf, which was 26% higher than the un-inoculated control.

**TABLE 6:** Effect of inoculation with multi-strain bacterial consortium on chlorophyll content (mg/g fresh weight) and membrane stability index of wheat at different salinity levels in a pot trial.

Strain	Chlorophyll content				Electrolyte leakage			
	Salinity (dS m^–1^)				Salinity (dS m^–1^)			
	2.1	6	12	18	2.1	6	12	18
T_1_	4.49 abc	4.14 def	2.65 g	1.11 i	41.3 f	40.7 f	46.6 def	55.16 bc
T_2_	4.56 ab	4.21 def	3.99 f	2.33 h	42.9 ef	41.9 ef	47.2 def	60.03 b
T_3_	4.50 abc	4.35 bcd	4.20 def	2.35 h	42.9 ef	44.1 ef	49.3 cde	56.80 bc
T_4_	4.62 a	4.29 cde	4.09 ef	2.52 gh	44.1 ef	43.4 ef	54.1 bcd	69.53 a
LSD (5%)	0.247				7.542			

Means sharing the same letter(s) are statistically non-significant according to Duncan’s multiple range test (p < 0.05).

T_1_ (Control), T_2_ (UA-46 + SN15), T_3_ (UA-3 + SN15 + UA-46), T_4_ (BK-30 + SN5 + SN15).

At a high salinity level of 17.5 dS m^–1^, the inoculated plants showed more proline contents compared to un-inoculated (Table 7). The plant inoculated with bacterial consortium T_4_ showed the highest proline contents (12% more than the un-inoculated control). However, the strains were statistically similar to each other. Similar to proline content, the inoculation effect regarding RWC was non-significant at low salinity levels (5.8 and 12.1 dS m^–1^). Up to a 19% increase in RWCs was recorded due to inoculation with different bacterial consortiums (Table 7). At a high salinity level (17.5 dS m^–1^), all three bacterial consortia significantly increased RWC over the un-inoculated control. Bacterial consortium T_4_ was the most effective for increasing RWC (19% more than the un-inoculated control).

**TABLE 7:** Effect of inoculation with multi-strain bacterial consortium on proline (μmole g^–1^) and relative water content of wheat at different salinity levels in a pot trial.

Strain	Proline				Relative water content			
	Salinity (dS m^–1^)				Salinity (dS m^–1^)			
	2.1	6	12	18	2.1	6	12	18
T_1_	0.70 e	0.86 d	1.17 c	1.26 bc	76.0 abc	68.5 de	62.1 fg	51.7 h
T_2_	0.64 e	0.99 d	1.18 c	1.39 ab	78.0 ab	70.2 cd	63.3 efg	59.1 g
T_3_	0.65 e	1.00 d	1.31 abc	1.41 a	78.4 a	72.0 bcd	62.8 efg	59.8 g
T_4_	0.64 e	0.94 d	1.31 abc	1.42 a	77.7 ab	71.8 bcd	66.4 def	61.4 ef
LSD (5%)	0.147				6.31			

Means sharing the same letter(s) are statistically non-significant according to Duncan’s multiple range test (p < 0.05).

T_1_ (Control), T_2_ (UA-46 + SN15), T_3_ (UA-3 + SN15 + UA-46), T_4_ (BK-30 + SN5 + SN15).

Data regarding sodium and potassium revealed that inoculation with bacterial consortium had variable effects on Na^+^ and K^+^ uptake by wheat plants. A significant decrease in Na^+^ concentration in the leaf sap at high salinity levels was observed due to inoculation with bacterial strains. However, more K^+^ concentration was observed in the leaf sap in inoculated treatments compared to the un-inoculated control, which resulted in a high K^+^/Na^+^ ratio in inoculation treatments compared to the un-inoculated control (Table 8). At a high salinity level (17.5 dS m^–1^), the highest increase in K^+^/Na^+^ was observed by inoculation with consortium T_4_ that was 31% more than the un-inoculated control.

**TABLE 8:** Effect of inoculation with multi-strain bacterial consortium on K/Na of wheat at different salinity levels in a pot trial.

Strain	Salinity (dS m^–1^)			
	2.1	6	12	17
T_1_	1.95 cd	1.90 d	1.60 gh	1.30 j
T_2_	2.11 b	1.98 cd	1.66 fgh	1.46 j
T_3_	2.23 a	2.04 c	1.80 e	1.67 fgh
T_4_	1.99 c	2.06 c	1.73 efg	1.57 hi
LSD (5%)	0.11			

Means sharing the same letter(s) are statistically non-significant according to Duncan’s multiple range test (p < 0.05).

T_1_ (Control), T_2_ (UA-46 + SN15), T_3_ (UA-3 + SN15 + UA-46), T_4_ (BK-30 + SN5 + SN15).

Data regarding nitrogen contents of wheat grain showed that inoculation significantly increased the nitrogen content at low as well as high salinity levels; however, strains had variable effects (Table 9). Up to a 17% increase in nitrogen content was observed with T_4_ at low salinity levels (5.8 dS m^–1^). At 12.1 and 17.5 dS m^–1^, T_3_ and T_4_ caused a maximum increase in nitrogen content, respectively. The wheat straw sample analyzed for phosphorus showed variable effects on the phosphorus contents of wheat grain (Table 9). At low-salinity levels (original and 5.8 dS m^–1^), inoculation with consortium T_4_ caused a maximum increase in phosphorus contents. At 12.1 and 17.5 dS m^–1^, inoculation showed the non-significant difference. Inoculation with bacterial consortium T_4_ showed maximum phosphorus contents (64% more than the un-inoculated control), which were statistically similar with T_2_ and T_3_.

**TABLE 9:** Effect of inoculation with multi-strain bacterial consortium on nitrogen and phosphorus content (%) of wheat grain at different salinity levels in a pot trial.

Strain	Nitrogen				Phosphorus			
	Salinity (dS m^–1^)				Salinity (dS m^–1^)			
	2.1	6	12	18	2.1	6	12	18
T_1_	1.23 cd	1.20 cd	1.17 de	0.91 f	0.23 c-g	0.22 efgh	0.20 fghi	0.14 j
T_2_	1.60 ab	1.22 cd	1.22 cd	1.26 cd	0.27 bcd	0.26 bcd	0.23 d-h	0.19 ghi
T_3_	1.53 ab	1.22 cd	1.41 bc	1.11 de	0.30 ab	0.27 bcde	0.24 c-g	0.18 hi
T_4_	1.69 a	1.41 bc	1.31 cd	1.30 cd	0.32 a	0.28 abc	0.24 cdef	0.23 d-h
LSD (5%)	0.199				0.052			

Means sharing the same letter(s) are statistically non-significant according to Duncan’s multiple range test (p < 0.05).

T_1_ (Control), T_2_ (UA-46 + SN15), T_3_ (UA-3 + SN15 + UA-46), T_4_ (BK-30 + SN5 + SN15).

## Discussion

Salinity is a major constraint for agricultural production, especially in arid and semi-arid regions of the world. The problem of salinity is also increasing due to the use of poor quality of underground water because of the non-availability of canal water. There are various approaches to combat the problem of salinity. The biological approach used in this study is also one of the emerging approaches. Although a number of studies have been conducted for promoting plant growth through PGPR inoculation; however, due to inconsistent results of single bacterial strain, the multi-strains consortium has been made to reduce the uncertainties related to this approach.

The results of the salinity tolerance assay showed that all strains were not equally effective to maintain their growth in saline environments. Some strains maintain their growth even at high concentrations of salts. This ability of a strain to maintain its growth at high salt concentration might be due to some of its particular mechanisms. These include the production of osmolytes and polysaccharides like exopolysaccharides. For example, to maintain growth in a saline environment, the bacteria establish and develop their internal pressure above the surrounding environment, and they generally achieve this by the accumulation of osmolytes in their body (Rhodes and Hanson, 1993). The survival of *Pseudomonas aeruginosa* in the sea by the accumulation of glycine and betaine is an example of this mechanism (Bakhrouf et al., 1991). Betaine is also involved in the biosynthesis of cyclopropane fatty acids to increase the membrane stability of *Pseudomonas halosaccharolytica* under extreme salty conditions (Monteoliva-Sanchez et al., 1993). Similarly, the production of exopolysaccharides may also protect the microbes from sodium toxicity, because these polysaccharides have the ability to bind Na^+^ and thus reduced their mobility (Upadhyay et al., 2011). Exopolysaccharide’s production character of bacterial strain is an important characteristic that protects the plant from the negative impact of stress. Shultana et al. (2020) observed that salt-tolerant PGPR strains containing exopolysaccharides caused a positive effect on the growth and yield of rice grown on salt-affected soils.

For better performance of bacterial strains when these are used in combination, the compatibility of strains with each other is a pre-requisite so better results can be achieved. In certain cases, a multi-strain microbial consortium is prepared by mixing microbes without taking antagonistic interactions that occur among the strains and therefore reduces their efficacy (Sarma et al., 2015). In this study, before preparing a multi-strain consortium, bacterial strains were checked for their compatibility with each other. It has been observed that some strains did not show compatibility with each other. This might be due to the reason for the production of certain compounds like cyanide that cause a negative impact on the growth of living organisms. The production of cyanide is a common characteristic of certain bacterial strains (Bakker and Schippers, 1987). Keeping in view the drawback of non-compatible strains, only the compatible strains were evaluated for their ability to promote plant growth under stress conditions.

Results of jar and pot trials indicated that the growth of wheat was affected by salinity stress. The effect of different salinity levels was variable on the growth parameters of wheat. All the growth parameters of wheat, i.e., shoot length, root length, shoot fresh weight, shoot dry weight, root fresh weight, and root dry weight were decreased due to salinity stress. Hindrance in plant growth and development in saline conditions might be due to the osmotic effect of salts and/or due to the toxic effect of salts within the plant (Munns and Tester, 2008). A decrease in plant growth might also be attributable to impaired mineral nutrient absorption and their translocation within the plant (Glenn et al., 1999). Moreover, root growth might reduce due to excessive biosynthesis of stress-induced ethylene (Mayak et al., 2004; Nadeem et al., 2007, 2010; Ahmad et al., 2013). Ethylene biosynthesis is increased due to salinity stress, which might be a key factor in the inhibition of root growth (Zahir et al., 2012). All these factors may be collectively responsible for decreased growth of wheat in salinity stress. All the growth parameters of wheat were improved due to inoculation with PGPR. This improvement in the growth of wheat at all salinity levels very likely be due to one or more growth-promoting activities of selected PGPR strains. But, most importantly this increase in growth of wheat seedlings in salinity stress might be due to the lowering of salinity-induced biosynthesis of ethylene in roots by ACC-deaminase activity of PGPR strains, which ultimately resulted in more root length (Glick et al., 1999; Mayak et al., 2004). In general, biosynthesis of ethylene is regulated by PGPR-containing ACC-deaminase, and they maintain its concentration below the growth inhibition level (Glick et al., 2007). The improvement in shoot length and shoot fresh and dry weight under salinity stress might be due to this increase in root length because an extensive root system is primarily important for better plant growth and development as plant vigor is directly related to the better root system (Doty et al., 2007). Furthermore, root and shoot growth of wheat might increase due to a decline in the concentration of Na^+^ ion within the plant before toxic limits by inoculation with PGPR strains possibly due to exopolysaccharides activity which may bind it and decrease its availability for plant uptake (Ashraf et al., 2004), as PGPR strains were positive for exopolysaccharide production. Moreover, Zhang et al. (2008) reported that bacterial inoculation with *Bacillus subtilis* GB03 decreases Na^+^ concentration within plants due to tissue-specific down- and upregulation of high-affinity K^+^ transporter (HKT1) in roots and shoots, respectively, which controls Na^+^ uptake and transport in plants. The better growth of wheat under salinity stress might also be a result of better protection against oxidative stress by PGPR-containing ACC-deaminase activity (Upadhyay et al., 2012).

In this study, salinity stress caused a negative impact on physiological parameters of wheat including chlorophyll content, electrolyte leakage, proline content, and RWCs. It has been observed that salinity enhanced the electrolyte leakage; however, a significant decrease in electrolyte leakage was observed in treatments where a multi-strain consortium was applied. Similarly, increasing the order of salinity caused a significant decrease in RWC. Other studies also mentioned the decrease in RWC in the presence of salinity (Yildrim et al., 2008; Yasin et al., 2018). The application of bacterial strains alone or in combination mitigated this negative impact of salinity relative to water content; however, the effect was more pronounced where multi-strain microbial consortium was applied. The increase in RWC and decrease in electrolyte leakage at varying levels of salinity has been observed by other workers by the application of effective strains of bacteria (Shultana et al., 2020).

Salinity also negative impact on chlorophyll contents of wheat and this effect was more pronounced at high salinity concentration. It has already been described by various researchers that salinity causes a decline a chlorophyll contents that ultimately affect the photosynthesis rate of the plant (Aquino et al., 2007; Silva et al., 2019). However, in present study, it has been observed that under salinity stress, the application of multi-strain bacterial consortium was found to be an effective strategy to improve the photosynthetic pigments that ultimately improve the photosynthesis rate of the plant. Sapre et al. (2018) also reported an increase in chlorophyll content of oat seedlings under salinity stress due to the application of *Klebsiella* sp.

Proline is a biochemical marker of salinity stress. Proline accumulation plays an osmoregulatory role to protect the plant from salt stress (Kavi Kishor et al., 2005). A multi-strain bacterial consortium of *Bacillus* sp. *Azospirillum brasilense, and Azosprillum lippferum* improved the biomass of wheat. The consortium also decreases electrolyte leakage and enhanced the chlorophyll content, RWC, proline content, amino acid, and antioxidant enzymes Akhtar et al. (2021).

A study conducted by Numan et al. (2018) demonstrated that improved plant growth under salinity stress was due to stimulation of osmoprotectants utilizing the microbial population for inducing salt tolerance rice. They further reported that it was due to ACC deaminase activity of inoculated strain, better root colonization, and enhancing chlorophyll and proline content. *Bacillus spp*. alleviated the harmful impact of salinity on pepper and improved growth by inducing salinity tolerance through the accumulation of proline (Wang et al., 2018). Similarly, He et al. (2018) also reported the more accumulation of proline content in *Haloxylon amodendron* inoculated ryegrass that induces salinity tolerance in said plant.

It is a well-documented effect that a high concentration of Na^+^ not causes a toxic effect on plant growth but also decreases the uptake of other essential nutrients, therefore, causing nutritional imbalances (Hirpara et al., 2005; da Costa and de Medeiros, 2018; Bekmirzaev et al., 2020). Sodium (Na^+^) and potassium (K^+^) play a major role in plant physiology under salinity stress. A high K/Na ratio is considered as one of the prerequisites for plant salinity tolerance. In this study, it has been observed that a significant decrease in K^+^ content occurred under salinity stress, particularly in un-inoculated treatments. Maximum Na content was observed in un-inoculated control treatments. Shahzad et al. (2019) also reported a high concentration of Na in plant tissue with an increase in salinity levels. The application of a multi-strain bacterial consortium mitigates this negative effect on K uptake. A decrease in Na uptake and an increase in K content have been observed that result in a high K/Na ratio. Other workers also reported the reduced uptake of Na^+^ and enhanced uptake of K^+^ by the application of bacterial strains (Khan et al., 2019). Recently, Shultana et al. (2020) while working on rice varieties having different salt tolerant abilities observed that inoculation with *Bacillus* spp. caused a significant decrease in the Na/K ratio under saline conditions.

Better plant growth is dependent on the availability of nutrients (Glick, 1995) and better uptake of essential plant nutrients, such as N, P, and K, with PGPR application, play an important role in plant growth (Hassan et al., 2015 p. 38). Multi-strain microbial inoculation also improved the plant nutrition by enhancing the uptake of nitrogen, phosphorus, and potassium. The improvement in the physiological parameters of the plant could be the reason for the enhanced uptake of essential nutrients. This better nutrition of wheat in salinity stress could be due to the production of siderophores (Singh R. K. et al., 2022) by these PGPR. Other reason could be the production of exopolysaccharides that play a significant role in biofilm formation that enhances the bacterial colonization on plant root (Chen et al., 2013) and restrict Na^+^ under saline conditions (Ashraf and Harris, 2004). Therefore, the ability of microbial strains to bind Na^+^ due to the production of exopolysaccharides decreases the availability of Na^+^ near the root environment, which ultimately reduces the antagonistic effect of Na^+^ on other essential plant nutrients.

Comparatively, better growth of wheat in salinity stress due to co-inoculations and multi-strain inoculation might be owing to improved colonization potential and sustainability of inoculants as compared to single strain inoculation (Raja et al., 2006). This is more likely to be successful survival and maintenance of different PGPR strains in communities (Andrews, 1991). Microbial studies performed without plants indicated that some combinations allow the bacteria to interact with each other synergistically, provide nutrients, remove inhibitory products, and stimulate each other through physical and biochemical activities that may enhance some beneficial aspects of their physiology (Bashan, 1998). Rajasekar and Elango (2011) studied the effectiveness of *Azospirillum*, *Azotobacter*, *Pseudomonas*, and *Bacillus* separately and in combination on the ashwagandha plant (*Withania somniferous*) for two consecutive years. They observed that PGPR consortia significantly increased plant height, root length, and alkaloid content in *Withania somnifera* when compared to the un-inoculated control and single inoculation. Jha et al. (2012) observed that growth of Jatropha plant improved maximally in greenhouse and field experiments when four strains were applied together. Co-inoculation provides the largest and most consistent increases in shoot weight, root weight, total biomass, shoot and root length, total chlorophyll, shoot width, and grain yield. In certain soil conditions, single strain inoculation does not provide the desired result due to the reason that inoculating strain could not compete with soil microflora. Poor root colonization and survival efficiency as well as soil factors, such as pH, temperature, humidity, etc., affect the performance of inoculating strain (Elkoca et al., 2010). However, in the case of a multi-strain microbial consortium, owing to their compatibility with each other, the microbial strain communicates synergistically with each other and enhances plant growth and development (Wani et al., 2007) as has been observed in this study.

## Conclusion

On an overall basis, it can be concluded that no doubt the single inoculation also caused a significant increase in wheat under salinity stress; however, this improvement was further enhanced due to the application of multi-strains inoculum. These preliminary studies that are conducted under laboratory and greenhouse conditions show the significant effectiveness of multi-strain compared to single strain inoculation; however, the effectiveness of this multi-strains consortium in a natural soil environment will further validate the results.

## Data availability statement

The data presented in this study can be found in the article/Supplementary material.

## Author contributions

MK, SN, ZZ, and FA-B contributed to conception and design of the study. MK, MS, MW, and LA organized the database. MK, FA, and MS performed the statistical analysis. MK and SN wrote the first draft of the manuscript. FA and MS wrote sections of the manuscript. All authors contributed to manuscript revision, read, and approved the submitted version.

## Footnotes

1
http://www.ncbi.nlm.nih.gov/BLAST
