# Supplementary material for: Potential of plant growth promoting bacterial consortium for improving the growth and yield of wheat under saline conditions
Source: Front Microbiol. 2022 Sep 29;13:958522. doi: 10.3389/fmicb.2022.958522 (PMC9557047; doi:10.3389/fmicb.2022.958522)
Supplement: Supplementary file 1 [file Table_1.docx]

**Supplementary Table 1.** **Effect of inoculation on shoot length (cm plant^-1^) of wheat in the presence of salinity (****d Sm^-1^) under axenic conditions**

| **Isolate** | **0.45 d Sm^-1^** | **6 d Sm^-1^** | **12 d Sm^-1^** | **18 d Sm^-1^** |
| --- | --- | --- | --- | --- |
| **Control** | 17.8±2.10 | 15.2±1.74 | 13.0±1.65 | 8.5±1.44 |
| **DG-8** | 20.4±2.10 | 17.9±2.00 | 18.0±1.56 | 16.4±1.46 |
| **DG-18** | 24.5±1.94 | 20.3±1.89 | 21.0±1.78 | 20.4±1.58 |
| **DG-26** | 26.4±1.87 | 17.3±1.74 | 17.7±1.70 | 16.7±1.58 |
| **DG-32** | 20.5±2.11 | 19.4±1.89 | 16.3±1.77 | 16.0±1.59 |
| **DG-34** | 25.7±1.88 | 20.2±1.88 | 18.7±1.75 | 18.5±1.80 |
| **DG-72** | 18.8±1.74 | 16.7±1.96 | 14.7±1.96 | 15.2±1.75 |
| **BK-6** | 20.5±1.99 | 21.4±1.89 | 20.0±1.78 | 17.9±1.49 |
| **BK-30** | 20.0±2.11 | 19.5±1.94 | 19.7±1.75 | 18.9±1.80 |
| **BK-33** | 19.5±2.00 | 17.2±2.00 | 18.0±1.74 | 16.5±1.56 |
| **BK-40** | 22.6±2.00 | 20.4±2.10 | 15.0±1.78 | 15.4±1.53 |
| **BK-46** | 23.5±1.96 | 20.2±1.89 | 19.3±1.81 | 18.5±1.60 |
| **BK-50** | 24.8±1.86 | 21.7±1.84 | 20.0±1.82 | 19.2±1.72 |
| **UA-1** | 24.1±1.88 | 22.5±1.88 | 16.0±1.83 | 15.5±1.66 |
| **UA-3** | 24.4±1.87 | 21.5±1.89 | 22.0±1.84 | 20.4±1.65 |
| **UA-39** | 19.8±1.89 | 17.4±1.88 | 16.7±1.99 | 14.3±1.70 |
| **UA-44** | 20.6±1.89 | 21.5±1.78 | 17.7±1.56 | 16.5±1.72 |
| **UA-46** | 23.4±1.84 | 22.4±1.74 | 20.0±1.59 | 18.5±1.73 |
| **UC-12** | 25.4±2.00 | 23.3±2.00 | 19.3±1.87 | 19.5±1.71 |
| **UC-14** | 18.7±1.96 | 18.5±1.89 | 17.0±1.74 | 16.5±1.50 |
| **UC-16** | 20.9±1.84 | 19.5±1.99 | 18.0±1.84 | 19.5±1.47 |

SED standard error of difference between two means; Average of four replicates ± standard error

**Supplementary Table 2.** **Effect of inoculation on shoot fresh weight (g plant^-1^) of wheat in the presence of salinity (dSm^-1^) under axenic conditions**

| **Isolate** | **0.45 d Sm^-1^** | **6 d Sm^-1^** | **12 d Sm^-1^** | **18 d Sm^-1^** |
| --- | --- | --- | --- | --- |
| **Control** | 0.94±0.18 | 0.84±0.14 | 0.75±0.12 | 0.46±0.14 |
| **DG-8** | 1.01±0.17 | 0.85±0.16 | 0.78±0.15 | 0.64±0.10 |
| **DG-18** | 0.98±0.18 | 0.93±0.18 | 0.82±0.16 | 0.70±0.14 |
| **DG-26** | 1.02±0.15 | 0.92±0.14 | 0.84±0.18 | 0.66±0.09 |
| **DG-32** | 0.97±0.19 | 0.86±0.14 | 0.79±0.19 | 0.67±0.18 |
| **DG-34** | 1.00±0.20 | 0.87±0.15 | 0.83±0.20 | 0.70±0.16 |
| **DG-72** | 1.01±0.18 | 0.93±0.17 | 0.85±0.24 | 0.66±0.11 |
| **BK-6** | 1.01±0.17 | 0.90±0.18 | 0.83±0.26 | 0.62±0.15 |
| **BK-30** | 1.04±019 | 0.93±0.17 | 0.81±0.21 | 0.71±0.14 |
| **BK-33** | 0.96±0.19 | 0.92±0.16 | 0.83±0.20 | 0.67±0.14 |
| **BK-40** | 1.00±0.20 | 0.92±0.15 | 0.85±0.15 | 0.60±0.16 |
| **BK-46** | 1.03±0.23 | 0.91±0.16 | 0.84±0.14 | 0.72±0.14 |
| **BK-50** | 0.96±0.22 | 0.95±0.17 | 0.87±0.20 | 0.71±0.10 |
| **UA-1** | 0.97±0.28 | 0.94±0.16 | 0.89±0.16 | 0.59±0.12 |
| **UA-3** | 0.98±0.24 | 0.88±0.14 | 0.83±0.15 | 0.74±0.11 |
| **UA-39** | 1.02±0.26 | 0.87±0.18 | 0.80±0.13 | 0.61±0.15 |
| **UA-44** | 1.08±0.28 | 0.93±0.16 | 0.86±0.18 | 0.69±0.14 |
| **UA-46** | 0.96±0.20 | 0.89±0.17 | 0.82±0.17 | 0.72±0.17 |
| **UC-12** | 1.10±0.19 | 0.93±0.19 | 0.85±0.16 | 0.72±0.18 |
| **UC-14** | 1.06±0.21 | 0.90±0.18 | 0.83±0.15 | 0.60±0.16 |
| **UC-16** | 0.96±0.23 | 0.92±0.15 | 0.87±0.14 | 0.71±0.15 |

SED standard error of difference between two means; Average of four replicates ± standard error

**Supplementary Table 3.** **Effect of inoculation on shoot dry weight (g plant^-1^) of wheat in the presence of salinity (dS m^-1^) under axenic conditions**

| **Isolate** | **0.45 d Sm^-1^** | **6 d Sm^-1^** | **12 d Sm^-1^** | **18 d Sm^-1^** |
| --- | --- | --- | --- | --- |
| **Control** | 0.34±0.08 | 0.26±0.09 | 0.19±0.07 | 0.11±0.06 |
| **DG-8** | 0.56±0.04 | 0.39±0.06 | 0.33±0.06 | 0.29±0.03 |
| **DG-18** | 0.48±0.0.9 | 0.41±0.04 | 0.31±0.05 | 0.30±0.04 |
| **DG-26** | 0.57±0.08 | 0.42±0.05 | 0.33±0.06 | 0.26±0.04 |
| **DG-32** | 0.51±0.06 | 0.39±0.06 | 0.21±0.04 | 0.24±0.03 |
| **DG-34** | 0.48±0.05 | 0.42±0.04 | 0.34±0.08 | 0.30±0.05 |
| **DG-72** | 0.59±0.12 | 0.45±0.09 | 0.29±0.09 | 0.21±0.02 |
| **BK-6** | 0.54±0.15 | 0.33±0.10 | 0.30±0.07 | 0.29±0.01 |
| **BK-30** | 0.54±0.09 | 0.30±0.07 | 0.30±0.07 | 0.31±0.05 |
| **BK-33** | 0.52±0.12 | 0.31±0.05 | 0.30±0.06 | 0.28±0.02 |
| **BK-40** | 0.53±0.11 | 0.29±0.04 | 0.27±0.08 | 0.26±0.03 |
| **BK-46** | 0.59±0.15 | 0.34±0.07 | 0.31±0.09 | 0.30±0.03 |
| **BK-50** | 0.49±0.10 | 0.33±0.08 | 0.41±0.09 | 0.32±0.02 |
| **UA-1** | 0.47±0.11 | 0.41±0.07 | 0.31±0.10 | 0.28±0.02 |
| **UA-3** | 0.49±0.12 | 0.46±0.07 | 0.33±0.10 | 0.30±0.04 |
| **UA-39** | 0.52±0.12 | 0.41±0.05 | 0.34±0.11 | 0.28±0.04 |
| **UA-44** | 0.51±0.10 | 0.41±0.06 | 0.36±0.09 | 0.29±0.04 |
| **UA-46** | 0.59±0.09 | 0.41±0.05 | 0.37±0.08 | 0.31±0.05 |
| **UC-12** | 0.56±0.11 | 0.44±0.06 | 0.38±0.06 | 0.32±0.02 |
| **UC-14** | 0.57±0.14 | 0.47±0.08 | 0.38±0.05 | 0.28±0.01 |
| **UC-16** | 0.55±0.12 | 0.46±0.08 | 0.35±0.05 | 0.33±0.02 |

SED standard error of difference between two means; Average of four replicates ± standard error

**Supplementary Table 4.** **Effect of inoculation on root length (cm plant^-1^) of wheat in the presence of salinity (dS m^-1^) under axenic conditions**

| **Isolate** | **0.45 d Sm^-1^** | **6 d Sm^-1^** | **12 d Sm^-1^** | **18 d Sm^-1^** |
| --- | --- | --- | --- | --- |
| **Control** | 16.7±1.78 | 12.8±1.56 | 9.5±1.40 | 6.3±1.09 |
| **DG-8** | 20.1±1.70 | 17.5±1.50 | 15.7±1.14 | 11.0±1.01 |
| **DG-18** | 19.8±1.80 | 17.1±1.50 | 19.0±1.15 | 17.5±1.07 |
| **DG-26** | 19.2±1.81 | 18.3±1.52 | 18.5±1.19 | 12.8±1.08 |
| **DG-32** | 16.8±1.80 | 16.7±1.57 | 15.2±1.40 | 10.5±1.10 |
| **DG-34** | 20.4±1.80 | 18.8±1.68 | 16.5±1.130 | 16.2±1.07 |
| **DG-72** | 22.6±1.79 | 15.8±1.60 | 13.7±1.16 | 13.4±1.11 |
| **BK-6** | 18.7±1.77 | 19.9±1.64 | 18.8±1.19 | 13.9±1.20 |
| **BK-30** | 20.2±1.59 | 19.5±1.70 | 14.5±1.29 | 16.7±1.18 |
| **BK-33** | 18.2±1.66 | 16.5±1.60 | 16.0±1.27 | 15.5±1.07 |
| **BK-40** | 17.6±1.78 | 17.5±1.61 | 13.3±1.30 | 14.7±1.09 |
| **BK-46** | 19.2±1.89 | 21.4±1.60 | 16.7±1.23 | 14.5±1.07 |
| **BK-50** | 18.9±1.88 | 18.7±1.70 | 16.5±1.24 | 14.3±1.09 |
| **UA-1** | 19.7±1.89 | 17.2±1.65 | 13.7±1.29 | 12.8±1.09 |
| **UA-3** | 21.2±2.00 | 16.9±1.66 | 15.0±1.30 | 14.8±1.10 |
| **UA-39** | 21.4±1.88 | 19.8±1.66 | 16.7±1.35 | 12.5±1.10 |
| **UA-44** | 19.3±1.91 | 18.5±1.60 | 16.4±1.40 | 13.5±1.10 |
| **UA-46** | 20.4±1.89 | 19.2±1.60 | 17.3±1.15 | 15.8±0.09 |
| **UC-12** | 18.9±1.99 | 18.2±1.58 | 15.3±1.18 | 13.4±0.09 |
| **UC-14** | 18.4±1.76 | 17.4±1.54 | 16.5±1.17 | 12.8±1.03 |
| **UC-16** | 20.4±1.71 | 16.9±1.60 | 15.3±1.16 | 15.1±1.02 |

SED standard error of difference between two means; Average of four replicates ± standard error

**Supplementary Table 5.** **Effect of inoculation on root fresh weight (g plant^-1^) of wheat in the presence of salinity (dS m^-1^) under axenic conditions**

| **Isolate** | **0.45 d Sm^-1^** | **6 d Sm^-1^** | **12 d Sm^-1^** | **18 d Sm^-1^** |
| --- | --- | --- | --- | --- |
| **Control** | 0.95±0.09 | 0.74±0.09 | 0.65±0.07 | 0.42±0.04 |
| **DG-8** | 0.99±0.08 | 0.75±0.06 | 0.71±0.06 | 0.56±0.04 |
| **DG-18** | 1.01±0.07 | 0.78±0.05 | 0.76±0.05 | 0.57±0.04 |
| **DG-26** | 1.02±0.08 | 0.79±0.07 | 0.78±0.05 | 0.53±0.04 |
| **DG-32** | 0.99±0.08 | 0.75±0.07 | 0.70±0.06 | 0.51±0.04 |
| **DG-34** | 0.98±0.08 | 0.93±0.07 | 0.77±0.06 | 0.61±0.06 |
| **DG-72** | 0.98±0.07 | 0.77±0.05 | 0.68±0.04 | 0.48±0.03 |
| **BK-6** | 1.01±0.06 | 0.72±0.07 | 0.68±0.05 | 0.53±0.03 |
| **BK-30** | 1.07±0.08 | 0.82±0.05 | 0.77±0.04 | 0.58±0.03 |
| **BK-33** | 1.05±0.05 | 0.85±0.05 | 0.77±0.04 | 0.54±0.03 |
| **BK-40** | 0.96±0.06 | 0.77±0.05 | 0.68±0.04 | 0.56±0.03 |
| **BK-46** | 0.98±0.06 | 0.81±0.05 | 0.79±0.04 | 0.50±0.03 |
| **BK-50** | 1.03±0.06 | 0.82±0.05 | 0.75±0.04 | 0.59±0.04 |
| **UA-1** | 1.04±0.05 | 0.96±0.05 | 0.72±0.04 | 0.53±0.02 |
| **UA-3** | 1.05±0.07 | 0.90±0.05 | 0.73±0.04 | 0.57±0.04 |
| **UA-39** | 0.99±0.08 | 0.82±0.05 | 0.78±0.04 | 0.56±0.03 |
| **UA-44** | 1.01±0.07 | 0.78±0.06 | 0.74±0.05 | 0.52±0.04 |
| **UA-46** | 1.02±0.07 | 0.88±0.06 | 0.74±0.05 | 0.58±0.03 |
| **UC-12** | 1.05±0.09 | 0.95±0.06 | 0.73±0.05 | 0.58±0.02 |
| **UC-14** | 1.04±0.07 | 0.83±0.06 | 0.76±0.04 | 0.47±0.03 |
| **UC-16** | 1.01±0.07 | 0.77±0.06 | 0.71±0.04 | 0.60±0.03 |

SED standard error of difference between two means; Average of four replicates ± standard error

**Supplementary Table 6. Effect of inoculation on root dry weight (g plant^-1^) of wheat in the presence of salinity (dSm^-1^) under axenic conditions**

| **Isolate** | **0.45 d Sm^-1^** | **6 d Sm^-1^** | **12 d Sm^-1^** | **18 d Sm^-1^** |
| --- | --- | --- | --- | --- |
| **Control** | 0.40±0.10 | 0.30±0.07 | 0.15±0.03 | 0.09±0.02 |
| **DG-8** | 0.50±0.11 | 0.34±0.06 | 0.40±0.02 | 0.22±0.02 |
| **DG-18** | 0.49±0.10 | 0.33±0.07 | 0.33±0.02 | 0.24±0.01 |
| **DG-26** | 0.48±0.10 | 0.36±0.06 | 0.33±0.03 | 0.15±0.02 |
| **DG-32** | 0.48±0.10 | 0.32±0.05 | 0.34±0.04 | 0.17±0.02 |
| **DG-34** | 0.51±0.10 | 0.35±0.05 | 0.36±0.02 | 0.24±0.03 |
| **DG-72** | 0.51±0.07 | 0.35±0.04 | 0.33±0.04 | 0.18±0.02 |
| **BK-6** | 0.57±0.10 | 0.30±0.03 | 0.36±0.03 | 0.20±0.02 |
| **BK-30** | 0.50±0.07 | 0.39±0.06 | 0.33±0.03 | 0.24±0.02 |
| **BK-33** | 0.51±0.10 | 0.31±0.08 | 0.31±0.04 | 0.21±0.01 |
| **BK-40** | 0.51±0.10 | 0.32±0.04 | 0.31±0.02 | 0.19±0.02 |
| **BK-46** | 0.49±0.09 | 0.28±0.04 | 0.31±0.03 | 0.21±0.02 |
| **BK-50** | 0.48±0.08 | 0.39±0.08 | 0.35±0.03 | 0.23±0.03 |
| **UA-1** | 0.47±0.07 | 0.34±0.04 | 0.34±0.03 | 0.18±0.02 |
| **UA-3** | 0.42±0.08 | 0.35±0.08 | 0.32±0.04 | 0.23±0.03 |
| **UA-39** | 0.42±0.10 | 0.31±0.07 | 0.33±0.05 | 0.17±0.03 |
| **UA-44** | 0.43±0.10 | 0.41±0.06 | 0.31±0.03 | 0.20±0.01 |
| **UA-46** | 0.49±0.10 | 0.41±0.05 | 0.34±0.04 | 0.28±0.01 |
| **UC-12** | 0.49±0.19 | 0.42±0.04 | 0.32±0.03 | 0.22±0.02 |
| **UC-14** | 0.51±0.08 | 0.42±0.06 | 0.29±0.04 | 0.19±0.02 |
| **UC-16** | 0.53±0.09 | 0.44±0.06 | 0.37±0.05 | 0.24±0.02 |

SED standard error of difference between two means; Average of four replicates ± standard error
